# Brain metastasis in children with stage 4 neuroblastoma after multidisciplinary treatment

**DOI:** 10.1186/s40880-015-0038-2

**Published:** 2015-09-14

**Authors:** Jia Zhu, Juan Wang, Zi-Jun Zhen, Su-Ying Lu, Fei Zhang, Fei-Fei Sun, Peng-Fei Li, Jun-Ting Huang, Rui-Qing Cai, Xiao-Fei Sun

**Affiliations:** State Key Laboratory of Oncology in South China, Department of Pediatric Oncology, Collaborative Innovation Center for Cancer Medicine, Sun Yat-sen University Cancer Center, Guangzhou, 510060 Guangdong P. R. China

**Keywords:** Neuroblastoma, Post treatment, Brain metastasis, Stage 4

## Abstract

**Introduction:**

Brain metastasis is common in relapsed neuroblastoma patients, but the characteristics of brain metastasis remain largely unknown. This study aimed to investigate the status of brain metastasis with neuroblastoma in South China.

**Methods:**

In this retrospective case-based study, 106 patients with stage 4 neuroblastoma from the Department of Pediatric Oncology in Sun Yat-sen University Cancer Center between January 2004 and May 2013 were included. The incidence, risk factors, and survival status of these patients were reviewed and analyzed.

**Results:**

Of the 106 patients, 11 (10.4%) developed brain metastasis, accounting for 20.0% of 55 patients with relapse or progression. The age at initial diagnosis of the 11 patients ranged from 2 to 10 years (median 4 years), which was younger than that of the patients without brain metastasis (median 5 years, range 1–10 years, *P* = 0.073). The male to female ratio of the 11 patients was 8:3, which was not significantly different from that of the patients without brain metastasis (*P* = 0.86). Patients with brain metastasis had higher lactate dehydrogenase levels than those without brain metastasis, but the differences were not significant (*P* = 0.076). Eight patients died, and 3 patients survived. The median interval from the initial diagnosis to the development of brain metastasis was 18 months (range 6–32 months). The median survival was 4 months (range 1 day to 29 months) after the diagnosis of brain metastasis. The median interval from the manifestation of brain metastasis to death was 3 months (range 1 day to 11 months).

**Conclusions:**

High-risk factors for brain metastasis in cases of neuroblastoma include bone marrow involvement and a younger age at initial diagnosis. Nevertheless, multiple treatment modalities can improve disease-free survival.

## Introduction

Neuroblastoma is a type of extracranial solid tumor that is most commonly observed in children. Neuroblastoma cells arise from the adrenal medulla or paraspinal sites where sympathetic nervous system tissue is located. The heterogeneous biological behavior of this tumor varies in terms of clinical course. Their broad spectrum of clinical behavior ranges from spontaneous regression to differentiation to benign ganglioneuroblastoma or ganglioneuroma, or to aggressive invasion with metastases to the liver, bone, bone marrow, and central nervous system (CNS). Approximately 60% of patients with stage 4 neuroblastoma achieve complete remission with multidisciplinary treatment, which involves chemotherapy, surgery, radiation, hematopoietic stem cell transplantation, and maintenance therapy including immunotherapy [1]. Although the survival of patients with high-risk neuroblastoma has improved [2], most patients eventually died from relapse. Relapsed neuroblastoma metastasizes to the CNS as one cause of death. The overall incidence of brain metastasis in neuroblastoma after treatment ranges from 1.7% to 11.7% [3].

No systemic study of metastasis to the CNS as the main site of progression or recurrence in neuroblastoma patients has been reported in China. We retrospectively analyzed the disease progression, recurrence, and metastases to the brain parenchyma, intraventricular areas, and medulla oblongata in patients with relapsed neuroblastoma at the Department of Pediatric Oncology in Sun Yat-sen University Cancer Center (SYSUCC). Our study aimed to gain insight into the incidence, risk factors, and prognosis of neuroblastoma patients with brain metastasis after multidisciplinary treatment.

## Patients and methods

### Patient selection

The clinical data of eligible patients with stage 4 neuroblastoma who were treated at the Department of Pediatric Oncology in SYSUCC between January 2004 and May 2013 were collected. These patients met the following criteria: (a) the patients with stage 4 neuroblastoma were younger than 18 years at the initial diagnosis; (b) brain metastasis was not observed at initial diagnosis; (c) the patients achieved complete response (CR) or partial response (PR) or had stable disease (SD) at the primary sites after multidisciplinary treatment according to the International Neuroblastoma Response Criteria [4]; and (d) the patients experienced the first occurrence of brain involvement after they achieved CR, PR or SD. The patients with primary brain tumors and those who had intracranial leptomeningeal metastasis or direct extension of tumors from the adjacent skull or dura mater were excluded.

### *MYCN* status and staging

The *MYCN* amplification was detected by fluorescence in situ hybridization in neuroblastoma samples after 2008. The staging evaluation was performed according to the International Neuroblastoma Staging System (INSS) [5]. Any primary neuroblastoma with dissemination to distant lymph nodes, the bone, bone marrow, liver, skin, and/or other organs was classified as stage 4 tumor.

### Definition of brain metastasis

The diagnosis of brain metastasis was based on clinical characteristics and magnetic resonance imaging (MRI) results if cerebral biopsy was infeasible. The clinical symptoms of patients with brain metastasis included headache and vomiting. The MRI of brain metastasis showed that intracranial metastases were localized to the parenchyma, ventricles, leptomeninges, and medulla oblongata [6]. Pathologic diagnosis of brain metastasis was performed by detecting the surgical specimens.

### Treatment

Patients with high-risk stage 4 neuroblastoma, received multidisciplinary treatment including chemotherapy, surgery, radiotherapy, autologous stem cell transplant (ASCT), and maintenance therapy including immunotherapy.

#### Chemotherapy

Four to six cycles of induction chemotherapy were administered prior to surgery. First-line chemotherapy consisted of vincristine 1.5 mg/m^2^ on day 1, cyclophosphamide 1.2–1.5 g/m^2^ on day 1, and doxorubicin 50 mg/m^2^ on day 1 (CAV regimen), or cisplatinum 20 mg/m^2^ on days 1–5 and etoposide 100 mg/m^2^ on days 1–5 (EP regimen)/etoposide 100 mg/m^2^ on days 1–4 and ifosfamide 1500 mg/m^2^ on days 1~4 and cisplatinum 20 mg/m^2^ on days 1~4 (VIP regimen), which were alternately administered at 3-week intervals. CAV and EP regimen were alternately administered at 3-week intervals before 2010. CAV and VIP regimen were alternately administered at 3-week intervals after 2010. 

#### Surgery

An evaluation of extensive disease was performed after induction chemotherapy. Patients underwent surgery to remove the primary tumor as completely as possible. Evaluation of the extent of the tumor excision was based on the Shamberger criteria [7]; the tumors were classified into three types: complete resection without macroscopic residual tumor, subtotal resection with ≤20% residual tumor, and partial resection with 21%–50% residual tumor.

#### Radiotherapy

Radiotherapy was performed as consolidation therapy after chemotherapy and surgery. The target volume was delineated according to the imaging examination and surgery documentation. The target volume included the primary lesion. The lymphatic drainage area was covered if lymph node involvement was suspected. The prescribed doses were 1.8–2.0 Gy/d by using 6.0–8.0 MV X-ray and 18.0–40.0 Gy in total (median 25.6 Gy) with intensity-modulated radiation therapy (IMRT) [7].

#### Autologous stem cell transplant (ASCT)

ASCT was performed in the patients with CR before 2008. The conditioning regimen included melphalan, carboplatin and etoposide.

#### Maintenance therapy

13-cis-retinoic acid was administered to high-risk patients following the completion of consolidation therapy. The dosage of 13-cis-retinoic was 100–160 mg/m^2^ in two divided doses for consecutive 7 days every 14 days, lasting 1 year.

Immunotherapy consisting of amplified human leukocyte antigen (HLA)-haploidentical donor immune cell infusion, cytokine-induced killer (CIK) cells, or natural killer (NK) cells was administered to selected patients with minimal residual disease or those who achieved CR after completion of chemotherapy, surgery, and radiotherapy [1]. One patient received anti-GD2 antibody immunotherapy in Hong Kong University.

#### Salvage therapy

When brain metastasis or recurrence occurred, salvage therapy including surgery, radiotherapy, and chemotherapy was administered.

Salvage radiotherapy: Patient 5 received radiotherapy 2 months after surgery. Patient 4 received radiotherapy 20 days after surgery. The radiotherapy for other patients is shown in Table 1.

**Table 1:** Treatment strategies and outcomes of 8 patients with brain metastasis

Patient no.	Metastatic site	Surgery	Radiotherapy	Chemotherapy	Outcome
1	Bilateral cerebral hemisphere, right temporal lobe	No	32.4 Gy	IFO + VM26, 4 cycles	Died
2	Right frontal lobe	Partial resection	25.4 Gy	TMZ + CPT-11, 2 cycles; VIP, 5 cycles	Died
3	Bilateral frontal lobes	No	25.2 Gy	No	Died
4	Right temporal lobe	Complete resection	27.0 Gy whole brain cranial irradiation + 30.0 Gy localized irradiation	CPT-11 + TMZ, 6 cycles; CPT-11 + Bevacizumab, 1 cycle	Currently alive for 47 months
5	Right frontal lobe, left cerebellum, left frontal lobe, spinal cord	Partial resection of the right frontal lobe	30.0 Gy localized irradiation + 30.0 Gy spinal cord irradiation	CPT-11 + TMZ, 2 cycles; CPT-11 + Nedaplatin + VCR, 5 cycles	Currently alive for 29 months
6	Left cerebellum, medulla	No	30.0 Gy localized irradiation	CPT-11 + VCR + TMZ, 4 cycles; VIP, 1 cycle	Still alive for 30 months
7	Left frontal lobe	No	No	CPT-11 + TMZ, 1 cycle	Died
8	Left parietal lobe	No	No	CPT-11 + TMZ, 2 cycles; CPT-11 + Bevacizumab, 1 cycle	Died

*IFO* ifosfamide, *VM26* teniposide, *TMZ* temozolomide, *CPT-11* irinotecan, *VIP* etoposide, ifosfamide, and cisplatinum, *VCR* vincristine.

Salvage chemotherapy: Six patients were treated with 2 cycles (range 1–6 cycles) of chemotherapy that consisted of temozolomide and irinotecan at 3-week intervals; a combination of vincristine 1.5 mg/m^2^ on day 1, irinotecan 20 mg/m^2^ on days 1–5, and temozolomide 100 mg/m^2^ on days 1–5 administered 1 h before irinotecan.

### Follow-up

Follow-up was performed by telephone, by mail, and in the clinic. The date of the last follow-up was December 31, 2013. The follow-up period was calculated from the time of initial diagnosis to death or the last follow-up, whichever occurred first.

### Statistical analysis

The Kaplan–Meier method was used to calculate the overall survival (OS) and event-free survival (EFS). A Chi square test was used to compare variables in two dependent groups, and the Wilcoxon test was used to compare the age distribution between the presence and absence of brain metastasis. A value of *P* < 0.05 was considered statistically significant.

## Results

### Patient characteristics and outcomes after initial treatment

A total of 106 patients with stage 4 neuroblastoma were involved in this study. The patients comprised 70 boys and 36 girls, with a median age of 6 years (range 1–18 years).

Of the 106 children, 81 (76.4%) achieved CR, PR, or SD after multidisciplinary treatments. Twelve patients with CR received ASCT. After the completion of multidisciplinary treatment, all patients underwent maintenance therapy and were therefore followed up. The 5-year OS rate in patients who achieved the first CR or good PR was 45.9%. Of the 81 patients, 55 developed disease relapse and progression, including 11 patients who developed brain metastasis (Table 2).

**Table 2:** Comparison of the clinical characteristics between relapsed patients with and without brain metastasis

Variable	Patients with brain metastasis	Patients without brain metastasis	*P* value
Total (cases)	11	44	
Age (years)			
Median	4	5	0.073
Range	2–10	1–10	
Sex (cases)			
Male	8	30	0.632
Female	3	14	
*MYCN* status (cases)^a^			
Positive	2	6	0.572
Negative	5	11	
Bone marrow involvement (cases)			
Yes	11	35	0.101
No	0	9	
Lactate dehydrogenase^b^			
Median (U/L)	568	353	0.076
Range (U/L)	197–3,906	188–3,903	
0–500 U/L (cases)	6	23	0.533
501–1,000 U/L (cases)	4	10	
>1,000 U/L (cases)	1	9	
Tumor response (cases)			
CR	8	34	0.719
PR	1	6	
SD	2	4	

*CR* complete response, *PR* partial response, *SD* stable disease.

^a^For *MYCN* status, 4 of 11 patients with brain metastasis and 27 of 44 patients without brain metastasis had no available data.

^b^Of 44 patients without brain metastasis, 2 had no data of lactate dehydrogenase. Two and ten patients in group with brain metastasis and without brain metastasis respectively underwent autologous stem cell transplant.

Of the 11 patients whose brains were the initial site of recurrence or progression, 8 were boys and 3 were girls, with a median age of 4 years (range 2–10 years). Of the other 44 patients with initial relapse or progression at other sites, 30 were boys and 14 were girls, with a median age of 5 years (range 1–10 years). The overall incidence of CNS metastasis was 10.4% (11/106) in patients with stage 4 neuroblastoma and was 20.0% (11/55) in patients with disease relapse or progression (Table 2).

The 11 patients who developed brain metastases after multidisciplinary treatment had bone marrow involvement at the initial diagnosis; two of 11 received ASCT at initial treatment; in addition, the *MYCN* amplification was detected in two of these patients. After multidisciplinary treatment before brain metastasis occurred, 8 patients achieved CR, 1 achieved PR, and 2 had SD. The median interval from the initial diagnosis to the development of brain metastasis was 18 months (range 6–32 months). The frontal and temporal lobes were the most common sites of brain recurrence or metastasis, as observed in 10 of 11 patients. One patient had concurrent cerebellar metastasis, and 1 had metastasis to the medulla oblongata.

### Outcomes of 11 neuroblastoma patients with metastasis to the CNS after treatment

Of the 11 patients with CNS metastasis, 3 died at 1, 18, and 30 days, respectively, after giving up treatment; 8 received salvage therapy as shown in Table 1.

Of the 8 patients who were treated with salvage chemotherapy, 2 patients with *MYCN* amplification died: 1 died 3 months after the time of the detection of brain metastasis, and 1 died 10 months after the detection of brain metastasis; of the 5 patients without *MYCN* amplification, 3 were still alive at the last follow-up, and 2 died. One patient with unknown *MYCN* status died (Table 1).

### Survival time from CNS metastasis

After a median follow-up time of 24 months, 8 of the 11 patients died from tumor progression. The remaining 3 patients were alive for 29, 30, and 47 months, respectively, after the initiation of multidisciplinary therapy. The median OS was 25 months (range 9–47 months) for the 11 patients. The median interval from the initial diagnosis to the development of brain metastasis was 18 months (range 6–32 months). The median survival after the development of metastases in the CNS was 4 months (range 1 day to 29 months).

## Discussion

Neuroblastoma is the most common extracranial solid tumor in children. Although multidisciplinary treatment including chemotherapy, surgery, radiotherapy, ASCT, and maintenance therapy with 13-cis-retinoic acid can induce the complete remission of high-risk neuroblastoma, most patients die from relapse and metastasis. Metastasis to the brain, which is a site of relapse or progression, is one such cause of death. In this study, we analyzed the incidence, clinical characteristics, and prognosis of brain metastases in cases of neuroblastoma in the absence of anti-GD2 monoclonal antibodies.

Our study indicated that the incidence of brain metastasis in patients with high-risk neuroblastoma was 10.4% (11/106). For the high-risk neuroblastoma patients who achieved a first CR or PR or had SD, the incidence of brain metastasis was 20.0% (11/55). All of the patients with brain metastases had bone marrow involvement. The median age of the 11 patients with brain metastases was younger than the age of the patients without brain metastasis. Three patients were alive at 29, 30, and 47 months, respectively, after the initiation of multidisciplinary therapy. The median OS was 25 months (range 9–47 months) for the 11 patients, and the median interval from the initial diagnosis to the development of brain metastasis was 18 months (range 6–32 months). The median survival after the development of CNS metastasis was 4 months (range 1 day to 29 months). Metastatic sites were located primarily in the frontal and temporal lobes.

The cooperative clinical trials in advanced countries have shown that the overall incidence of CNS metastasis increased from 1.7% to 11.7% in patients who were treated with protocols from N4-N5 to N6-N7 and had prolonged survival [3]. With respect to chemotherapy, the Memorial Sloan-Kettering Cancer Center uses cycles of cisplatin, etoposide, cyclophosphamide, doxorubicin, and vincristine to treat patients with high-risk and metastatic neuroblastoma [8]. Post-N4 and post-N5 consolidation therapy consists of high-dose melphalan- or thiotepa-based chemotherapy with autologous bone marrow rescue [9]. The consolidative treatment with N6 and N7 protocols involves immunotherapy with the anti-GD2 murine monoclonal antibody 3F8, either alone or labeled with ^131^I. The survival of patients with high-risk neuroblastoma increased to >38%. Of the 127 newly diagnosed patients with stage 4 neuroblastoma, 8 (6.3%) developed isolated CNS metastasis [9]. Our cancer center treated patients with high-risk neuroblastoma with similar chemotherapy protocols but without the anti-GD2 murine monoclonal antibody. In our study, the survival of patients was improved, but brain metastasis occurred more frequently. Paulino et al. [6] reported that brain metastases developed in 8% (9 of 113) of neuroblastoma patients. In our study, 11 patients with stage 4 neuroblastoma developed brain metastases after receiving multidisciplinary treatment and had bone marrow involvement but had not brain metastasis at the initial diagnosis. The incidence of brain metastasis in our center was slightly higher than that in other developed countries. High-intensity chemotherapy and ASCT failed to prevent CNS relapse or progression, although high-intensity chemotherapy prolonged the survival of high-risk patients with neuroblastoma [10, 11]. Among our patients with brain metastasis, 2 patients received ASCT at initial therapy and 1 case also received anti-GD2 antibody in Hong Kong University after ASCT. These patients still relapsed and had brain metastasis. It was similar to the report from Choi [11] that 4/11 had brain metastasis after CD34(+) transplantation.

The risk factors associated with the development of brain metastasis in neuroblastoma patients are controversial. Lumbar puncture, high levels of serum lactate dehydrogenase (LDH), and orbital metastases at the time of diagnosis were associated with brain metastasis [8]. Based on the limited number of patients in our center, age and bone marrow involvement at initial diagnosis were associated with the subsequent development of brain metastasis, although the difference was not statistically significant between the groups with and without brain metastasis. We hypothesized that the hematogenous spread from infiltrated bone marrow may break through the immature blood–brain barrier (BBB) and develop into brain metastases. The level of LDH has also been determined to be a prognostic factor [10]. A high level of serum LDH was found to represent in the patients with a higher tumor burden. Kramer et al. [8] found that elevated serum LDH (>1,500 U/mL) was also a significant risk factor for brain metastasis. The difference in LDH levels was not statistically significant between the two groups with and without brain metastasis in our study. It may be that the patients with higher levels of serum LDH died prior to the development of brain metastasis. Our results indicated no significant difference in *MYCN* amplification between the groups with and without brain metastasis. Further studies will be necessary to test the role of *MYCN* amplification in the development of brain metastasis. The *MYCN* amplification was associated with an advanced stage of neuroblastoma and rapid progression [12]. Some patients with *MYCN* amplification died before the development of brain metastasis. In our study, 2 patients with *MYCN* amplification died 3 months after brain metastasis, suggesting that *MYCN* amplification facilitates tumor growth and metastatic potential.

Traditionally, neuroblastoma patients with brain metastasis had a poor prognosis, and in China, parents of these patients tended to refuse further therapy once brain metastasis was diagnosed. Based on a set of limited cases, we found that patients with isolated CNS metastasis whose brain lesions were completely resected or demonstrated a good response to chemotherapy like other chemo-sensitive CNS tumors, for example CNS germ cell tumors [13], had a better prognosis and a prolonged survival. In our study, 3 patients with brain metastasis are alive after salvage therapy. Bone marrow infiltration of Patient 4 resolved soon after chemotherapy. He underwent complete removal of brain tumor, cranial radiotherapy 20 days following surgery and achieved a CR; his mental and motor statuses remained normal until the last follow-up, and he returned to school. The other 2 surviving patients underwent incomplete resection of the brain metastases and survived with tumors.

The combination of vincristine, temozolomide, and irinotecan (VIT) was effective against refractory neuroblastoma [14]. The regimen VIT can cross the BBB. If temozolomide is administered 1 h prior to irinotecan, the two agents have a synergetic antineoplastic activity for brain and bone marrow metastases. Irinotecan is a camptothecin prodrug, and its active metabolite (SN-38) inhibits the nuclear enzyme topoisomerase I. The temozolomide-induced formation of O^6^-methylguanine adducts alkylate or methylate DNA, and therefore it facilitates the creation of topoisomerase I/DNA complexes. The concentration of temozolomide in the serum reaches its peak 1 h after administration, suggesting that DNA-methylating agents could potentially augment the activity of camptothecins [14]. Four patients achieved PR after receiving VIT chemotherapy. Patient 4 responded well to chemotherapy with irinotecan, resulting in CR in the bone marrow and disease-free survival of 47 months. Kramer et al. [15] evaluated the addition of compartmental intrathecal antibody-based radioimmunotherapy (cRIT) in neuroblastoma patients with metastatic CNS neuroblastoma following surgery, craniospinal irradiation, and chemotherapy; finally, 17 of the 21 patients who were treated with cRIT salvage therapy were alive 7–74 months (median 33 months) after CNS relapse, and the remaining patients were free of CNS metastasis.

Our study had some limitations. The detection of *MYCN* status was not previously performed in all patients. Immunotherapy that targets the molecules expressed in neuroblastoma cells warrants further investigation and may be used to prevent brain metastasis.

## Conclusions

In summary, our study suggests that the incidence of brain metastasis increased in neuroblastoma patients with a prolonged survival. High-risk factors may include bone marrow involvement and a younger age at initial diagnosis. Patients with brain lesions that are completely resected and those with a CR to chemotherapy and radiotherapy in terms of resolution of bone marrow involvement may have a better prognosis.
